# Cigarette smoking, e-cigarette, and heated tobacco product use and associated factors among a nationally representative sample of adult Malaysians: An online survey

**DOI:** 10.18332/tid/221104

**Published:** 2026-06-27

**Authors:** Chandrashekhar T. Sreeramareddy, Fateen-Izzah Haziqah Parlan, Palanisamy Sivanandy, Pathiyil R. Shankar

**Affiliations:** 1 Division of Community Medicine and Public Health, IMU University, Kuala Lumpur, Malaysia; 2 Department of Pharmacy Practice, School of Pharmacy, IMU University, Kuala Lumpur, Malaysia; 3 IMU Centre for Education, IMU University, Kuala Lumpur, Malaysia

**Keywords:** tobacco, heated products, smoking, public, health

## Abstract

**INTRODUCTION:**

We aimed to estimate the prevalence of cigarette smoking, e-cigarette, and heated tobacco product use behaviors and their associated factors among adult Malaysians.

**METHODS:**

A cross-sectional online survey was conducted in December 2024 among a nationally representative sample of 1000 adult Malaysians (≥18 years). We used a questionnaire adapted from the Global Adult Tobacco Survey. Binary logistic regression analyses were performed to identify factors associated with tobacco and nicotine product use.

**RESULTS:**

The overall prevalence of current smoking was 28.6% (95% CI: 25.8–31.4), with a mean age of smoking initiation of 21.5 years. Awareness of e-cigarettes was high (92.4%), with 14.6% (95% CI: 12.5–17.0) and 13.4% (95% CI: 11.4–15.8) reporting current daily and non-daily use, respectively. Fewer respondents were aware of heated tobacco products (HTPs, 548.5%), with 4.5% (95% CI: 3.4–6.1) and 10.0% (95% CI: 8.2–12.1) reporting current daily and non-daily use, respectively. Current smoking (AOR=44.19; 95% CI: 24.78–78.81 for e-cigarettes; AOR=31.0; 95% CI: 13.58–73.57 for HTPs), male sex, younger age, and exposure to advertisements were significantly associated with e-cigarette and HTP use. A smoke-free home environment was a protective factor against e-cigarette (AOR=0.44; 95% CI: 0.24–0.80) and HTP use (AOR=0.27; 95% CI: 0.13–0.55).

**CONCLUSIONS:**

Continued surveillance of nicotine product use is essential. Tobacco control measures under the new Public Health Act should include specific regulations for e-cigarettes and heated tobacco products, particularly targeting younger populations.

## INTRODUCTION

Tobacco smoking is a leading cause of disease burden worldwide^1^. The tobacco-attributable burden is high in low- and middle-income countries due to the higher prevalence of tobacco use and larger populations^2^. In recent decades, the prevalence of tobacco smoking has been declining worldwide, albeit at a slower pace in LMICs^3^. Concurrently, the big tobacco companies have introduced newer tobacco products such as electronic cigarettes, heated tobacco products, and oral nicotine pouches, which are being marketed and sold as harm-reduction alternatives to cigarette smoking, particularly in high-income countries^4,5^. In recent years, many LMICs have seen an increase in e-cigarette and HTP users^6,7^. The Southeast Asian countries, particularly Indonesia, Malaysia, Vietnam, and the Philippines, are reported to have considerable markets for newer products, whereas the policy response to regulate the newer products is weak^8^.

Malaysia, an upper middle-income country, is going through a socioeconomic transition, has an aging population, and is a signatory to the World Health Organization Framework Convention on Tobacco Control (WHO-FCTC)^9^. Data from the serial National Health and Morbidity Survey reports have shown that current smoking prevalence in Malaysia has been stationary at about 25% since 1996^10^. However, NHMS does not collect data on e-cigarettes and HTPs^11^. Estimates of e-cigarette and HTP use rates are available for Malaysia from transnational tobacco surveys such as Global Adult Tobacco Surveys (GATS)^12^, and International Tobacco Control (ITC) surveys in Malaysia (2013/14)^13^. GATS Malaysia (2013) has reported prevalence estimates of awareness, ever, and current use of e-cigarettes^14^, and the ITC Survey 2020 has reported on e-cigarette and HTP use among the adult population^15,16^. However, up-to-date estimates of both cigarette smoking and newer non-cigarette nicotine products on a nationally representative sample of Malaysian adults are needed for monitoring to know the population-level impact of tobacco control measures^9^. Reports from LMICs have emphasized the need for surveillance of e-cigarettes and HTP use behaviors as well, to inform the regulatory policies^6,7^. The existing literature on nationally representative samples in Malaysia only reports the estimates of e-cigarette and HTP use and reasons for their use15-18. The association of e-cigarette and HTP use with factors related to tobacco control, such as knowledge about the health effects of smoking, exposure to information about the dangers of tobacco smoking, and advertisements about tobacco products, smoke-free policies at home are reported in previous studies^7,19^, but such studies are not reported from Malaysia.

Malaysia has introduced new tobacco control measures under the Control of Smoking Products for Public Health Act 2024 (Act 852), which came into effect in Malaysia on 1 October 2024^20^. In December 2024, we conducted an online survey to measure Malaysian public support for the existing tobacco control measures and the newly implemented Act 852. From the survey data, we aimed to provide the prevalence estimates of cigarette smoking, e-cigarette, and heated tobacco product use behaviors among adult Malaysians and determine the sociodemographic, pro- and anti-tobacco factors associated with tobacco and nicotine product use behaviors.

## METHODS

### Design, participants, and setting

A cross-sectional online survey of adult Malaysians (age ≥18 years) was conducted to obtain public opinion towards tobacco control policies in Malaysia. The survey covered all Malaysian states and territories.

### Sample size and sampling methodology

For 99% confidence, a finite population size (Malaysian population), 5% sampling error, and an anticipated proportion of 50% of adult Malaysians that support tobacco control strategies in Malaysia (50% since previous estimates for this proportion are unknown), are needed. Using the formula for estimating the population proportion, a sample of 652 was obtained. For an anticipated 30% non-response rate, a sample of 1000 was required. Stratified sampling was used to obtain a representative sample comparable to Malaysia’s latest population census data. The sample surveyed followed quotas for the region of residence, sex, and age groups to ensure the final sample surveyed was proportional to stratum sizes based on Malaysian census data.

### Study instrument

Our questionnaire was adapted from selected sections of the Global Adult Tobacco Survey (GATS)^12^. GATS is a household survey electronically administered using handheld tablets among non-institutionalized individuals aged ≥15 years. The questionnaire covered the participants’ demographics, tobacco use behaviors, including the use of electronic cigarettes and heated tobacco products (HTPs), knowledge about the health effects of tobacco smoking, exposure to information about the dangers of tobacco use, and advertisements about cigarettes, e-cigarettes, and heated tobacco products.

### Variables

The main outcome variables were tobacco use behaviors, classified according to the standard indicators of GATS. Current smoking, e-cigarette, and HTP use were based on the responses to the question: ‘Do you currently smoke tobacco, use e-cigarettes, and HTPs?’. Those who responded as daily or non-daily were coded as current e-cigarette/HTP use or current smoking. Ever use of e-cigarettes/HTPs was coded based on a ‘yes’ response to the question: ‘Have you ever used, even once, e-cigarettes or HTPs?’. Past smoking or use of e-cigarettes or HTPs was defined as those who smoked or had used e-cigarettes or HTPs in the past. Past smoking or past use of e-cigarettes or HTPs was coded based on a ‘yes’ response to the question: ‘Have you smoked or used e-cigarettes/HTPs in the past?’. The knowledge score about the health effects of tobacco smoking was computed by summing the ‘yes’ responses to the question: ‘Based on what you know or believe, does smoking cause the following’. A list of 12 health effects was provided with ‘yes’, ‘no’, and ‘do not know’ options. Exposure to information about the dangers of tobacco use and advertisements of cigarettes, e-cigarettes, and HTPs from different sources was computed by summing the ‘yes’ responses provided by respondents to the questions: ‘In the last 30 days, have you noticed information about the dangers of smoking cigarettes or that encourages quitting in any of the following places?’ (with options newspapers/magazines, television, radio, billboards, and internet) and ‘In the last 30 days, have you noticed any advertisements or signs promoting cigarettes in the following places?’ respectively. Ten options were provided for exposure to advertisements (Supplementary file).

### Data collection method

The survey was conducted by the survey research company Rakuten. All the surveys were completed online by the potential respondents who were verified during their registration. Launch was done through a blast of the survey link to the public while also monitoring the demographics quota to obtain a nationally representative sample of adults aged ≥18 years. The survey participant information sheet was sent out in the survey link; after reading the survey information, the potential participants were taken to the consent form, and upon checking it as yes, the full survey link was provided. Quality checks were constantly done to delete an incorrect response.

### Ethics and consent procedure

This study obtained ethical approval from the IMU University Joint Commission (IMU-JC) for research (project number 4.14/JCM-294/2024). Informed consent was obtained from all participants invited to participate in the survey. Participants were informed about the anonymity, and the study adhered to the principles of the Declaration of Helsinki.

### Data analysis

The categorical were summarized as frequencies and percentages, and the continuous variables as means and standard deviations (SD). Prevalence estimates and their 95% confidence intervals were calculated. To determine the factors associated with tobacco use behaviors, current smoking, e-cigarette, and HTP use (dependent variable), we carried out a binary logistic regression analysis. The independent variables were demographics, knowledge scores about the harms of cigarette smoking, rules about smoking at home, exposure to information about the dangers of smoking, and exposure to advertisements.

## RESULTS

### Demographics, smoking, e-cigarette, and HTP use behaviors

The mean age of the 1000 respondents (500 each male and female) who completed the survey was 38.7 years (SD=11.5), who were mainly of Malay race (60.0%), followed by Chinese. About half of them were working in the private sector (49.4%), while a quarter were not employed (housewives, retired, students, etc.). Nearly half of them were educated up to a Bachelor’s or higher degree (48.5%), and 61.5% were currently married (Table 1). The overall prevalence of current smoking (daily/non-daily) was 28.6% (95% CI: 25.8–31.4), past smoking was 7.9% (95% CI: 6.4–9.7), and the mean age at initiation of daily smoking was 21.5 years (SD=4.9) (Table 1). Most of the participants had heard about e-cigarettes (92.4%), and more than a third had ever (even once) used e-cigarettes. The prevalence of daily and non-daily e-cigarette use was 14.6% (95% CI: 12.5–17.0) and 13.4% (95% CI: 11.4–15.8), respectively. Only about half the respondents had heard about HTPs, and less than a fifth had ever (even once) used HTPs. The prevalence rates of daily and non-daily use of HTPs were 4.5% (95% CI: 3.4–6.1) and 10.0% (95% CI: 8.2–12.1), respectively (Table 2).

**Table 1:** Sociodemographic characteristics of adult participants in an online survey conducted in Malaysia, December 2024 (N=1000)

*Characteristics*	*n*	*%*
**Age** (years)		
18–34	238	23.8
35–44	462	46.2
≥45	300	30
**Gender**		
Male	500	50
Female	500	50
**Ethnicity**		
Malay	600	60
Chinese	250	25
Indian	100	10
Other	50	5
**Marital status**		
Single	384	38.4
Married	616	61.6
**Occupation**		
Private	494	49.4
Government	111	11.1
Self-employed	127	12.7
Unemployed	268	26.8
**Education level**		
Primary	242	24.2
Secondary	273	27.3
Undergraduate	485	48.5

**Table 2:** Cigarette smoking, e-cigarette, and heated-tobacco products use behaviors among adult participants in an online survey conducted in Malaysia, December 2024 (N=1000)

*Variables*	*% (95% CI)*
**Cigarette smoking**	
Current	28.6 (25.8–31.4)
Past	7.9 (6.4–9.7)
**Age at initiation** (years), mean	21.5 (20.97–22.14)
**E-cigarettes**	
Heard about them	92.4 (90.6–93.9)
Ever use (even once)	39.3 (36.2–42.5)
Current use (daily)	14.6 (12.5–17.0)
Current use (non-daily)	13.4 (11.4–15.8)
**Heated tobacco products**	
Heard about them	48.5 (45.4–51.6)
Ever use (even once)	18.3 (15.9–20.9)
Current use (daily)	4.5 (3.4–6.1)
Current use (non-daily)	10.0 (8.2–12.1)

### Comparison of smoking, e-cigarette, and HTP use behaviors by demographics

The prevalence rates of current smoking (44.2% vs 13.0%), current use of e-cigarettes (37.0% vs 14.0%), and HTPs (18.6% vs 8.6%) were significantly higher among men than women. E-cigarette (33.2% and 26.3%) and HTP (15.7% and 15.6%) use were significantly higher among the younger age groups (18–34 and 35–44 years) than those aged ≥45 years. Smoking was significantly higher among the Indians (37.0%), while HTP use was significantly higher among the Chinese (20.0%) than among the other races. HTP use was significantly higher among those with a Bachelor’s degree (16.4%). Smoking (34.5% and 36.8%), e-cigarette (29.9% and 33.4%), and HTP (15.7% and 18.0%) use were significantly higher among those who were self-employed and employed in the private sector. Smoking (32.1% vs 22.9%) and HTPs (15.9% vs 9.9%) were significantly higher among married individuals than single individuals. Smoking (32.8% and 72.0%), e-cigarette (34.3% and 63.9%), and HTP (18.9% and 40.3%) use were significantly higher among individuals who reported that smoking inside the home was allowed and not allowed, but with exceptions (Table 3).

**Table 3:** Distributions of current smoking, e-cigarette use, and HTP use by demographic characteristics of the adult participants in an online survey conducted in Malaysia, December 2024 (N=1000)

*Characteristics*	*Total n*	*Smoking*		*E-cigarettes*		*HTPs*	
		*n*	*%*	*n*	*%*	*n*	*%*
**Sex**							
Female	500	65	13.00	73	14.60	43	8.60
Male	500	221	44.20	186	37.20	93	18.60
**Age** (years)							
18–34	400	113	28.25	133	33.25	63	15.75
35–44	300	87	29.00	79	26.33	47	15.67
≥45	300	86	28.67	47	15.67	26	8.67
**Race**							
Other	50	8	16.00	7	14.00	2	4.00
Indian	100	37	37.00	24	24.00	12	12.00
Chinese	250	79	31.60	70	28.00	50	20.00
Malay	600	162	27.00	158	26.33	72	12.00
**Education level**							
Bachelor’s and higher	485	145	29.90	140	28.87	80	16.49
Secondary	273	80	29.30	65	23.81	35	12.82
Primary	242	61	25.21	54	22.31	21	8.68
**Marital status**							
Single	384	88	22.92		22.66	38	9.90
Married	616	198	32.14	172	27.92	98	15.91
**Employment**							
Unemployed	268	31	11.57	26	9.70	11	4.10
Self-employed	127	44	34.65	38	29.92	20	15.75
Government	111	29	26.13	30	27.03	16	14.41
Private	494	182	36.84	165	33.40	89	18.02
**Smoking rules at home**							
No rules	130	23	17.69	20	15.38	13	10.00
Never allowed	514	83	16.15	69	13.42	21	4.09
Not allowed but exceptions	195	64	32.82	67	34.36	37	18.97
Allowed	161	116	72.05	103	63.98	65	40.37

### Demographic and tobacco-related factors associated with smoking, e-cigarette, and HTP use

From binary logistic regression analyses, being a male, married, self-employed, and employed by the government, living in houses where smoking is not allowed with exceptions, and having no rules about smoking at home were associated with cigarette smoking. Men had a 6.2 times higher odds of smoking compared to females (AOR=6.21; 95% CI: 4.23–9.12); being married had a 1.9 times higher odds of smoking compared to being single (AOR=1.92; 95%CI: 1.28–2.87); being self-employed and employed in government had 1.9 and 2.3 times higher odds of being a smoker compared to those who were unemployed (AOR=1.91; 95% CI: 1.02–3.59) and (AOR=2.33; 95% CI: 1.21–4.52), respectively. Individuals from houses where smoking inside the house is not allowed with exceptions and allowed, had 2.5 and 12.5 times higher odds of smoking compared to individuals from houses where there were no rules about smoking inside the house (AOR=2.53; 95% CI: 1.38–4.63) and (AOR=12.46; 95% CI: 6.56–23.66), respectively (Table 4).

**Table 4:** Demographic, tobacco control-related factors associated with current cigarette smoking from binary logistic regression analysis

*Variables*	*AOR*	*95% CI*	*p*
**Age** (years)			
≥45 (ref.)	1		
35–44	0.86	0.55–1.34	0.503
18–34	0.95	0.606–1.50	0.839
**Sex**			
Female (ref.)	1		
Male	6.21	4.23–9.12	<0.001
**Race**			
Other (ref.)	1		
Indian	1.86	0.67–5.18	0.234
Chinese	1.49	0.57–3.88	0.419
Malay	1.053	0.43–2.60	0.91
**Education level**			
Bachelor’s and higher (ref.)	1		
Secondary	0.99	0.65–1.49	0.486
Primary	0.85	0.54–1.34	0.946
**Marital status**			
Single (ref.)	1		
Married	1.92	1.28–2.87	0.001
**Occupation**			
Unemployed (ref.)	1		
Self-employed	1.91	1.02–3.59	0.012
Government	2.33	1.21–4.52	0.001
Private	2.47	1.48–4.13	0.044
**Smoking rules at home**			
No rules (ref.)	1		
Never allowed	0.753	0.43–1.32	0.323
Not allowed, but exceptions	2.53	1.38–4.63	0.003
Allowed	12.46	6.56–23.66	<0.001
**Knowledge about the health effects of smoking**	0.96	0.91–1.02	0.158
**Exposure to information about the dangers of smoking**	1.037	0.92–1.17	0.542
**Exposure to advertisements for cigarettes**	1.025	0.96–1.09	0.437

AOR: adjusted odds ratio.

Current e-cigarette use was associated with age, smoking rules at home, and exposure to e-cigarette advertisements. Those aged 18–34 and 35–44 years, had 3.5 and 7.4 times higher odds of current e-cigarette use compared to those aged ≥45 years (AOR=3.50; 95% CI: 2.03–6.04) and AOR=7.44; 95% CI: 3.62–15.07), respectively. Individuals from households where smoking was not allowed (with exceptions) had 0.44 times lower odds of current e-cigarette use compared to those from houses in which there were no rules (AOR=0.44; 95% CI: 0.24–0.80). The odds of current use of e-cigarettes increased by a factor of 1.2 if media on which individuals had seen advertisements related to e-cigarettes (AOR=1.15; 95% CI: 1.05–1.25) (Table 5).

**Table 5:** Demographic, cigarette smoking, tobacco control-related factors associated with current e-cigarette and HTP use from binary logistic regression analysis

*Variables*	*E-cigarettes*			*HTPs*		
	*AOR*	*95% CI*	*p*	*AOR*	*95% CI*	*p*
**Age** (years)						
≥45 (ref.)	1			1		
35–44	3.50	2.03–6.04	<0.001	1.34	0.70–2.59	0.38
18–34	7.39	3.62–15.07	<0.001	1.11	0.48–2.59	0.806
**Sex**						
Female (ref.)	1			1		
Male	1.58	0.96–2.59	0.073	0.836	0.46–1.52	0.558
**Race**						
Other (ref.)	1			1		
Indian	1.18	0.33–4.22	0.795	2.18	0.29–16.53	0.45
Chinese	2.03	0.62–6.65	0.242	6.9	0.99–47.74	0.051
Malay	2.22	0.74–6.62	0.153	2.73	0.41–17.99	0.296
**Education level**						
Bachelor’s and higher (ref.)	1			1		
Secondary	0.64	0.37–1.09	0.1	0.62	0.33–1.16	0.132
Primary	0.78	0.45–1.36	0.38	0.42	0.20–0.87	0.02
**Marital status**						
Single (ref.)	1			1		
Married	1.35	0.80–2.28	0.26	1.81	0.97–3.39	0.063
**Occupation**						
Unemployed (ref.)	1			1		
Self-employed	1.45	0.67–3.15	0.343	1.34	0.48–3.78	0.58
Government	2.15	0.95–4.87	0.066	2.86	0.96–8.49	0.059
Private sector	1.73	0.90–3.30	0.099	1.23	0.50–3.03	0.648
**Smoking status**						
Never (ref.)	1			1		
Current	44.19	24.78–78.81	<0.001	31.61	13.58–73.57	<0.001
Past	9.10	4.46–18.57	<0.001	14.95	5.23–42.79	<0.001
**Smoking rules at home**						
No rules (ref.)	1			1		
Never allowed	1.00	0.53–1.90	0.994	1.139	0.58–2.23	0.704
Not allowed but exceptions	**0.44**	**0.24–0.80**	**0.007**	**0.267**	**0.13–0.55**	**<0.001**
Allowed	**0.48**	**0.21–1.07**	**0.073**	**0.87**	**0.35–2.14**	**0.761**
**Knowledge about the health effects of smoking**	1.03	0.96–1.10	0.468	1.04	0.96–1.13	0.363
**Exposure to information about the dangers of smoking**	0.95	0.80–1.13	0.572	**0.81**	**0.67–0.99**	0.036
**Exposure to information about e-cigarettes/HTPs**	1.12	0.96–1.32	0.162	1.23	1.01–1.50	0.046
**Exposure to advertisements for e-cigarettes/HTPs**	1.15	1.06–1.25	0.001	1.38	1.23–1.55	<0.001

AOR: adjusted odds ratio.

By binary logistic regression analyses, primary education, being from houses where smoking is not allowed (with exceptions), exposure to information about the dangers of smoking, and information about and advertisements of HTPs were associated with the current use of HTPs. The individuals with education up to primary level had 0.4 times lower odds of being current HTP users (AOR=0.42; 95% CI: 0.19–0.87) compared to those with a Bachelor’s or higher level of education. Individuals from households where smoking inside the house was never allowed had 0.27 times lower odds of current HTP use compared to those from households where there were no rules (AOR=0.27; 95% CI: 0.13–0.55). The odds of current HTP use decreased by a factor of 0.8 among individuals who reported seeing information about the dangers of smoking in the media (AOR=0.81; 95% CI: 0.67–0.99). The odds of current HTP use increased by a factor of 1.2 and 1.4 among the individuals who had seen information about HTPs and advertisements in the media (AOR=1.23; 95% CI: 1.01–1.50) and (AOR=1.38; 95% CI: 1.23–1.55), respectively. Compared to individuals who had never smoked, those who currently smoked had 44 times higher odds of current e-cigarette use and 31 times higher odds of current HTP use (AOR=44.19; 95% CI: 24.78–78.81) and (AOR=31; 95% CI: 13.58–73.57), respectively. Similarly, compared to individuals who had never smoked, those who had smoked in the past had 9 times higher odds of current e-cigarette use and 15 times higher odds of current HTP use (AOR=9.1; 95% CI: 4.46–18.57 and AOR=14.95; 95% CI: 5.23–42.79).

## DISCUSSION

Our online survey of a nationally representative sample of adults (≥18 years) provided comparable estimates of both cigarette smoking and nicotine product use behaviors in Malaysia. Online surveys showed that cigarette smoking is common among Malaysian adults. Awareness of e-cigarettes was very high, about a third had ever used an e-cigarette, and a third were currently using e-cigarettes. Higher proportions of e-cigarette users were those who smoked and had smoked in the past. About half of the respondents were aware of HTPs, about a fifth had ever used HTPs, and fewer currently used HTPs (mostly non-daily), mainly among those who smoked at present and smoked in the past. Cigarette smoking was associated with male sex, being married, self-employed/government employment, and a lack of restrictions or rules about smoking at home. Current e-cigarette use was associated with younger age, smoking rules at home, and exposure to advertisements on e-cigarettes. HTP use was associated with education, exposure to information about the dangers of smoking, and HTP advertisements.

Awareness about both e-cigarettes and HTPs was much higher than in other LMICs^6,7,19^. The prevalence estimates of current smoking in our online survey are higher than those of GATS and NHMS. Our estimates based on the sample of individuals surveyed online were comparable to those from another national-level face-to-face survey carried out in 2016^17^. Though the questions are similar, the higher estimates are perhaps due to the younger age of our sample, whereas it has been reported that in older age groups, prevalence is lower due to the cohort effect^21^. A higher prevalence of smoking among men, private and government employees, was comparable to NHMS reports^22^. NHMS reported a significantly higher prevalence of smoking among the less educated and Malay adults^22^. The sample surveyed in NMHS via a house-to-house survey versus the sample of adults active online would have likely resulted in such differences.

Estimates of e-cigarette and HTP use are comparable to those of the ITC online survey (2020)^15,18^. Our survey findings using the GATS questionnaire provided comparable estimates, which are necessary for surveillance of cigarette smoking prevalence towards the global tobacco endgame and Malaysian targets of 5% (2030) and 15% by 2030^23^. Estimates of e-cigarette use and HTP use are also important for the surveillance of these products, as they are not yet regulated in Malaysia. The e-cigarette use indicators have been on the rise since they were first reported in Malaysia in GATS 2011^14^. Ever since, there has been an increasing trend of e-cigarette use in Malaysia, as reported in other nationally representative sample surveys^18,24^. The prevalence of HTP use in our survey was comparable to ITC surveys^15^. Notably, our survey’s e-cigarette and HTP use rates were comparable to those of the ITC survey, which was also implemented by the same online survey company, Rakuten Insights. Since GATS, NHMS, and other national-level surveys used face-to-face methods and complex survey designs, they provided more representative samples for the Malaysian population^11,17,25^.

The pattern of a higher proportion of males compared to females, younger individuals, and higher educated individuals using e-cigarettes and HTPs was also observed in other Malaysian surveys^15,17,18^ and other LMICs^6,7,19^. Similarly, e-cigarette and HTP use among those who currently smoke cigarettes and smoked in the past was much higher than among those who had never smoked during their lifetime, as in other LMICs^6,7,19^ and Malaysia^17,18^. Overall, the pattern of e-cigarette use by demographic distribution and by smoking status is consistent with those from developed countries as well^26^. E-cigarette use showed that ever use of e-cigarettes/HTPs was associated with exposure to information about the dangers of tobacco use, and advertisements about e-cigarettes/HTPs in three GATS countries^7^. In our study, the current use of e-cigarettes and HTPs was associated with exposure to advertisements on nicotine products. Exposure to e-cigarette advertisements online and the use of e-cigarettes have been well-established^27^.

Rules about smoking inside the house were also associated with e-cigarette/HTP use^7^. Individuals from houses where smoking is not allowed (with exceptions) had lower odds of e-cigarette and HTP use. This is a plausible association since most nicotine product users were either smoking currently or had smoked in the past. The association of smoke-free homes with lower smoking rates has been reported before^19^. Our findings suggest that smoke-free policies at home should be encouraged to reduce e-cigarette/HTP use. There is a consistent association between e-cigarettes and HTP use with current and past smoking^15,17,18^. It is an important concern since dual use leads to higher addiction and health risks^28^. However, e-cigarette use among individuals who smoked in the past is safer, potentially not as hazardous; nevertheless, it may lead to the renormalization of tobacco use, posing challenges to the tobacco endgame^5^.

### Policy implications

Malaysia is a signatory to the World Health Organization Framework Convention for Tobacco Control^9^ and has implemented evidence-based interventions for tobacco control^29^. However, after a decade of stagnation of smoking prevalence at 25%^30^, the most recent GATS and NHMS have shown a small decline in smoking prevalence to about 20% in 2023 and 2024^25,31^. Despite a higher prevalence of smoking (28%) in our online survey, which is likely an overestimate, our findings are congruent with GATS and NHMS estimates^25,31^. Estimates of nicotine product use behaviors and their distribution by demographics and smoking status call for regulations on nicotine products, specifically among the younger populations of Malaysia. The challenge to this is the delisting of gel and liquid nicotine present in e-cigarettes from the ‘Poisons Act’ schedule in April 2023^32^. This measure officially legalizes e-cigarettes that contain nicotine, and the lack of regulations of HTPs in Malaysia is an obstacle to achieving the tobacco end game^5^. On 1 October 2024, Malaysia implemented the Control of Smoking Products for Public Health Act 2024 (Act 852), which mainly covers tobacco smoking^20^. The Public Health Act 852, however, does not detail any regulations on e-cigarettes and HTPs.

### Strengths and limitations

As the survey was based on the sample of adults who are active online and have participated in an online survey, the oversampling of younger, higher educated adults, perhaps from urban areas, could have resulted in higher estimates due to selection bias. Social desirability and report bias cannot be ruled out. The self-reported tobacco use behaviors were not validated with biomarker estimation; nevertheless, self-reported behaviors are known to be reliable^33^. Despite these limitations, our online survey provides estimates for surveillance of nicotine product use behaviors among young adults and those who have smoked cigarettes. Continued surveillance is critical to inform the regulatory policy.

## CONCLUSIONS

Our online survey of a nationwide sample of adults provided reliable and comparable estimates of cigarette smoking and nicotine product use behaviors among Malaysians. Continued surveillance of nicotine product use is needed through serial surveys among youth and young adults. Substantial rates of nicotine product use among the young, associated with exposure to advertisements, suggest the need for the implementation of regulations specific to nicotine products in addition to the Control of Smoking Products for Public Health Act.

## Supplementary Material



## Data Availability

The data supporting this research are available from the authors on reasonable request.

## References

[1] GBD 2019 Tobacco Collaborators. Spatial, temporal, and demographic patterns in prevalence of smoking tobacco use and attributable disease burden in 204 countries and territories, 1990-2019: a systematic analysis from the Global Burden of Disease Study 2019. Lancet. 2021;397(10292):2337-2360. doi:10.1016/S0140-6736(21)01169-7 34051883 PMC8223261

[2] World Health Organization. WHO report on the global tobacco epidemic, 2023: protect people from tobacco smoke. World Health Organization; 2023. Accessed March 19, 2026. https://www.who.int/publications/i/item/9789240077164

[3] Paraje G, Flores Muñoz M, Wu DC, Jha P. Reductions in smoking due to ratification of the Framework Convention for Tobacco Control in 171 countries. Nat Med. 2024;30(3):683-689. doi:10.1038/s41591-024-02806-0 38321222 PMC10957467

[4] O’Connor R, Schneller LM, Felicione NJ, Talhout R, Goniewicz ML, Ashley DL. Evolution of tobacco products: recent history and future directions. Tob Control. 2022;31(2):175-182. doi:10.1136/tobaccocontrol-2021-056544 35241585

[5] Bhatnagar A, Whitsel LP, Blaha MJ, et al. New and emerging tobacco products and the nicotine endgame: the role of Robust Regulation and Comprehensive Tobacco Control and Prevention: A Presidential Advisory from the American Heart Association. Circulation. 2019;139(19):e937-e958. doi:10.1161/CIR.0000000000000669 30862181

[6] Sreeramareddy CT, Manoharan A. Awareness about and e-cigarette use among adults in 15 low- and middle-income countries, 2014-2018 estimates from Global Adult Tobacco Surveys. Nicotine Tob Res. 2022;24(7):1095-1103. doi:10.1093/ntr/ntab269 35596725

[7] Sreeramareddy CT, Shu Syuen MO, Hon MI, Daher AM. Electronic cigarette and heated tobacco product use and their association with tobacco control factors among adults in Indonesia, Kazakhstan, and the Philippines. Nicotine Tob Res. 2025;27(2):254-261. doi:10.1093/ntr/ntae187 39110887

[8] van der Eijk Y, Tan Ping Ping G, Ong SE, et al. E-Cigarette Markets and Policy Responses in Southeast Asia: A Scoping Review. Int J Health Policy Manag. 2022;11(9):1616. doi:10.34172/ijhpm.2021.25 33906338 PMC9808234

[9] World Health Organization. Who Framework Convention On Tobacco Control. World Health Organization; 2003. Accessed March 19, 2026. https://iris.who.int/server/api/core/bitstreams/264104b3-241a-4e48-88f9-aa7120779ffc/content

[10] Teh CH, Rampal S, Lim KH, Azahadi O, Tahir A. Age, period, and cohort analysis of smoking intensity among current smokers in Malaysia, 1996-2015. Nicotine Tob Res. 2023;25(7):1340-1347. doi:10.1093/ntr/ntad035 36879440 PMC10256889

[11] Institute for Public Health Malaysia (IPH). The Third National Health and Morbidity Survey 2006 (NHMS III), Smoking. Ministry of Health, Malaysia; 2008. Accessed April 25, 2026. https://iku.nih.gov.my/images/IKU/Document/REPORT/2006/Smoking.pdf

[12] Palipudi KM, Morton J, Hsia J, et al. Methodology of the Global Adult Tobacco Survey - 2008-2010. Glob Health Promot. 2016;23(suppl 2):3-23. doi:10.1177/1757975913499800 24042975

[13] Thompson ME, Fong GT, Hammond D, et al. Methods of the International Tobacco Control (ITC) Four Country Survey. Tob Control. 2006;15(suppl 3):iii12-iii18. doi:10.1136/tc.2005.013870 16754941 PMC2593059

[14] Palipudi KM, Mbulo L, Morton J, et al. Awareness and current use of electronic cigarettes in Indonesia, Malaysia, Qatar, and Greece: Findings From 2011-2013 Global Adult Tobacco Surveys. Nicotine Tob Res. 2016;18(4):501-507. doi:10.1093/ntr/ntv081 25895951 PMC5100820

[15] Noh MAM, Hairi FM, Nordin ASA, et al. Prevalence and reasons for use of heated tobacco products among Malaysian adults who smoked: findings from the 2020 International Tobacco Control Malaysia Survey. Drug Alcohol Depend. 2024;257:111131. doi:10.1016/j.drugalcdep.2024.111131 38428371

[16] Hairi FM, Goh KT, Driezen P, et al. Reasons for using e-cigarettes and support for e-cigarette regulations: Findings from the 2020 ITC Malaysia Survey. Tob Induc Dis. 2022;20:33. doi:10.18332/tid/146364 35431720 PMC8969647

[17] Ab Rahman J, Mohd Yusoff MF, Nik Mohamed MH, et al. The prevalence of e-cigarette use among adults in Malaysia. Asia Pac J Public Health. 2019;31(suppl 7):9S-21S. doi:10.1177/1010539519834735 30880403

[18] Driezen P, Nordin ASA, Hairi FM, et al. E-cigarette prevalence among Malaysian adults and types and flavors of e-cigarette products used by cigarette smokers who vape: Findings from the 2020 ITC Malaysia Survey. Tob Induc Dis. 2022;20:32. doi:10.18332/tid/146363 35431719 PMC8969646

[19] Hussain S, Sreeramareddy CT. Smoking cessation behaviors and reasons for use of electronic cigarettes and heated tobacco products among Romanian adults. Sci Rep. 2022;12(1):5446. doi:10.1038/s41598-022-09456-7 35361852 PMC8968304

[20] Ministry of Health Malaysia. Laws Of Malaysia: Act 852: Control Of Smoking Products For Public Health Act 2024. Ministry of Health Malaysia; 2024. Accessed March 19, 2026. https://seatca.org/dmdocuments/Control%20of%20Smoking%20Products%20for%20Public%20Health%20Act%202024.pdf

[21] Lim KH, Jasvindar K, Cheong SM, et al. Prevalence of smoking and its associated factors with smoking among elderly smokers in Malaysia: findings from a nationwide population-based study. Tob Induc Dis. 2016;14:8. doi:10.1186/s12971-016-0073-z 27006650 PMC4802631

[22] Lim KH, Teh CH, Pan S, et al. Prevalence and factors associated with smoking among adults in Malaysia: Findings from the National Health and Morbidity Survey (NHMS) 2015. Tob Induc Dis. 2018;16:01. doi:10.18332/tid/82190 31516402 PMC6659615

[23] Ministry of Health Malaysia. National Strategic Plan For The Control Of Tobacco & Smoking Products 2021-2030; 2021. Accessed March 19, 2026. https://extranet.who.int/fctcapps/sites/default/files/2024-02/403_06102021-%28Final%29%20English-%20Pelan%20Strategik%20Kebangsaan%20English.pdf

[24] Gravely S, Driezen P, Ouimet J, et al. Prevalence of awareness, ever-use and current use of nicotine vaping products (NVPs) among adult current smokers and ex-smokers in 14 countries with differing regulations on sales and marketing of NVPs: cross-sectional findings from the ITC Project. Addiction. 2019;114(6):1060-1073. doi:10.1111/add.14558 30681215 PMC6510648

[25] National Institutes of Health, Ministry of Health, Malaysia. Global Adult Tobacco Survey (GATS): Malaysia 2023.; 2024. Accessed March 19, 2026. https://cdn.who.int/media/docs/default-source/ncds/ncd-surveillance/data-reporting/malaysia/mys-gats2023-factsheet.pdf

[26] Tehrani H, Rajabi A, Ghelichi-Ghojogh M, Nejatian M, Jafari A. The prevalence of electronic cigarettes vaping globally: a systematic review and meta-analysis [retracted In: Arch Public Health. 2024 Jul 26;82(1):113. doi: 10.1186/s13690-024-01345-x.]. Arch Public Health. 2022;80(1):240. doi:10.1186/s13690-022-00998-w 39061111 PMC11282610

[27] Luu NM, Phan TH, Oh JK, Myung SK. Exposure to electronic cigarette advertisements and use of electronic cigarettes: a meta-analysis of prospective studies. Nicotine Tob Res. 2023;25(5):983-990. doi:10.1093/ntr/ntac266 36426864

[28] Banks E, Yazidjoglou A, Brown S, et al. Electronic cigarettes and health outcomes: umbrella and systematic review of the global evidence. Med J Aust. 2023;218(6):267-275. doi:10.5694/mja2.51890 36939271 PMC10952413

[29] Lai PK, Nalliah S, Teng CL, Chen NLP. Translation of scientific evidence into tobacco control policy in Malaysia: A narrative review. Journal of Health and Translational Medicine (JUMMEC). 2022;25(1):89-96. doi:10.22452/jummec.vol25no1.15

[30] Mohd Yusoff MF, Lim KH, Saminathan TA, et al. The pattern in prevalence and sociodemographic factors of smoking in Malaysia, 2011-2019: Findings from national surveys. Tob Induc Dis. 2022;20:84. doi:10.18332/tid/152410 PMC9521184 36249344

[31] Ministry of Health Malaysia. National Health and Morbidity Survey 2023: Non-communicable Diseases and Healthcare Demand: Key Findings; 2024. Accessed March 19, 2026. https://iku.nih.gov.my/images/nhms2023/key-findings-nhms-2023.pdf

[32] CodeBlue. Malaysia Shockingly Legalises Vape With Nicotine For Tax– Without Existing E-Cigarette Regulations; 2023. Accessed March 19, 2026. https://codeblue.galencentre.org/2023/03/malaysia-shockingly-legalises-vape-with-nicotine-for-tax-without-existing-e-cigarette-regulations/#:~:text=Shockingly%2C%20the%20Excise%20Duties%20(Amendment,as%20the%20Poisons%20Board%20meeting

[33] Connor Gorber S, Schofield-Hurwitz S, Hardt J, Levasseur G, Tremblay M. The accuracy of self-reported smoking: a systematic review of the relationship between self-reported and cotinine-assessed smoking status. Nicotine Tob Res. 2009;11(1):12-24. doi:10.1093/ntr/ntn010 19246437

