# Supplementary material for: Cigarette smoking, e-cigarette, and heated tobacco product use and associated factors among a nationally representative sample of adult Malaysians: An online survey
Source: Tob Induc Dis. 2026 Jun 27;24:10.18332/tid/221104. doi: 10.18332/tid/221104 (PMC13312266; doi:10.18332/tid/221104)
Supplement: Supplementary file 1 [file TID-24-98-s1.pdf]

Awareness about and perception towards proposed Generational End Game among Malaysian general public: an online cross-sectional questionnaire study

PART 1: DEMOGRAPHIC/ DEMOGRAFIK

1. Age?/ Umur _____ years old/ tahun

2. Gender:
a. Male/ Lelaki
b. b. Female/ Perempuan

3. Ethnicity
a. Malay/ Melayu
b. Chinese/ Cina
c. Indian/ India
d. Others/ Lain-lain :_______

4. Marital Status
   a. Single/ Bujang
   b. Married/ Berkahwin
   c. Divorced/ Bercerai

5. Occupation/ Pekerjaan
   a. Private/ Swasta
   b. Government/ Kerajaan
   c. Self-employed/ Bekerja sendiri
   d. Unemployed/homemaker/ Menganggur/ suri rumah
   e. Retiree/ Pesara
   f. Other/ lain-lain: ____________(please state/ sila nyatakan)

6. Education level/ Tahap pendidikan
   a. Secondary/ Menengah
   b. Vocational/ Vokasional
   c. Diploma/ Diploma
   d. Undergraduate/ Sarjana muda
   e. Postgraduate/ Pascasiswazah
   f. Not schooling/ Tidak bersekolah

© 2026 Sreeramareddy C.T. et al.
DOI:10.18332/tid/221104

7. State/ Negeri:
a. Johor
b. Negeri Sembilan
c. Melaka
d. Pahang
e. Selangor
f. Perak
g. Terengganu
h. Kelantan
i. Kedah
j. Perlis
k. Pulau Pinang
l. Sabah
m. Sarawak

PART 2a: SMOKING STATUS/ STATUS MEROKOK

1. Do you currently smoke tobacco? Adakah anda merokok?
a. Daily basis/ Setiap hari
b. Less than daily/ Kurang dari setiap hari
c. Not at all/ Tidak sama sekali

If response is b or c move to question 2a. 3

2. How old were you when you first started smoking tobacco daily? (Write your answer) Berapa umur anda apabila anda mula menghisap tembakau setiap hari? (Sila nyatakan umur anda)

_________ years old/ tahun

3. In the past, have you smoked tobacco?
Pernahkan anda menghisap tembakau pada masa lalu?
a. Yes/ Ya
b. No/ Tidak

If response is b move to question 2a.5

4. Have you smoked tobacco **daily** in the past?
Pernahkan anda menghisap tembakau setiap hari pada masa lalu?
a. Yes/ Ya
b. No/ Tidak

© 2026 Sreeramareddy C.T. et al. DOI:10.18332/tid/221104

5. Prior to today, have you ever heard of electronic cigarettes?

Sebelum hari ini, adakah anda pernah mendengar tentang rokok elektronik?

   a. Yes/ Ya
   b. No/ Tidak

6. Have you ever, even once, used an electronic cigarette?

Pernahkah anda, walaupun sekali, menggunakan rokok elektronik?

   a. Yes/ Ya
   b. No/ Tidak

7. Do you currently use electronic cigarettes?

Adakah anda sedang menggunakan rokok elektronik?

   a. Daily basis/ Setiap hari
   b. less than daily/ Kurang dari sehari
   c. Not at all/ Tidak sama sekali

8. Have you ever used electronic cigarettes daily in the past?

Pernahkah anda menggunakan rokok elektronik setiap hari pada masa lalu?

   a. Yes/ Ya
   b. No/ Tidak

9. I want to ask you about products that heat but do not burn tobacco like iQOS and GLO. Prior to today, have you ever heard of heated tobacco products?

Saya ingin bertanya tentang produk yang memanaskan, tetapi tidak membakar tembakau seperti iQOS dan GLO. Sebelum hari ini, adakah anda pernah mendengar tentang produk tembakau yang dipanaskan?

   a. Yes/ Ya
   b. No/ Tidak

10. Have you ever, even once, used a heated tobacco product?

Pernahkah anda, walaupun sekali, menggunakan produk tembakau yang dipanaskan?

   a. Yes/ Ya
   b. No/ Tidak

11. Do you currently use heated tobacco products?

Adakah anda sedang menggunakan produk tembakau yang dipanaskan?

   a. Daily basis/ Setiap hari
   b. Less than daily/ Kurang dari sehari
   c. Not at all/ Tidak sama sekali

© 2026 Sreeramareddy C.T. et al. DOI:10.18332/tid/221104

12. Have you ever used heated tobacco products daily in the past?

Pernahkah anda menggunakan produk tembakau yang dipanaskan setiap hari pada masa lalu?

a. Yes/ Ya
b. No/ Tidak

PART 2b: SMOKING STATUS/ STATUS MEROKOK

1. Which of the following best describes the rules about smoking tobacco inside of your home?

Antara berikut, yang manakah menerangkan peraturan tentang menghisap tembakau di dalam rumah anda?

a. Allowed/ Dibenarkan
b. Not allowed but exceptions/ Tidak dibenarkan tetapi pengecualian
c. Never allowed/ Tidak pernah dibenarkan
d. No rules/ Tiada peraturan

2. Based on what you know or believe, does smoking cause the following:

Berdasarkan apa yang anda ketahui atau percaya, adakah merokok menyebabkan perkara berikut:

Disease/ Penyakit	Yes/ Ya	No/ Tidak	Don’t Know/ Tidak tahu
Stroke/ Strok			
Myocardial Infarction/ Infarksi miokardium			
Lung cancer/ Kanser paru-paru			
Emphysema/ Emfisema			
Stomach cancer/ Kanser perut			
Mouth cancer/ Kanser mulut			
Periodontal disease/ Penyakit gusi			
Bone loss/ Kerapuhan tulang			
Premature birth/ Kelahiran pramatang			
Erectile dysfunction/ Disfungsi erektil			
Tuberculosis/ Batuk kering			

© 2026 Sreeramareddy C.T. et al. DOI:10.18332/tid/221104

Diabetes/ Kencing manis			

3. In the last 30days, have you noticed information about the dangers of smoking **cigarettes** or that encourages quitting in any of the following places:

Dalam tempoh 30 hari yang lalu, adakah anda melihat maklumat tentang bahaya menghisap **rokok** atau menggalakkan berhenti **merokok** di mana-mana tempat berikut:

Places/ Tempat	Yes/ Ya	No / Tidak	Not Applicable/ Tidak berkaitan
Newspaper/ magazines Akhbar/ majalah			
Television/ Televisyen			
Radio/ Radio			
Billboards/ Papan iklan			
Internet/ Internet			
Others? Please specify:<br>Lain-lain? Sila nyatakan:<br>________________			

4. In the last 30days, have you noticed information about the dangers of **electronic cigarettes** or that encourages quitting in any of the following places:

Dalam tempoh 30 hari yang lalu, adakah anda melihat maklumat tentang bahaya menghisap **rokok elektronik** atau menggalakkan berhenti **rokok elektronik** di mana-mana tempat berikut:

Places/ Tempat	Yes/ Ya	No / Tidak	Not Applicable/ Tidak berkaitan
Newspaper/ magazines Akhbar/ majalah			
Television/ Televisyen			
Radio/ Radio			
Billboards/ Papan iklan			
Internet/ Internet			
Others? Please specify:<br>Lain-lain? Sila nyatakan:<br>________________			

5. In the last 30days, have you noticed information about the dangers of using **heated tobacco** products like iQOS or GLO in any of the following places:

© 2026 Sreeramareddy C.T. et al. DOI:10.18332/tid/221104

Dalam tempoh 30 hari yang lalu, adakah anda melihat maklumat tentang bahaya menggunakan **produk tembakau yang dipanaskan** seperti iQOS atau GLO di mana-mana tempat berikut:

Places/ Tempat	Yes/ Ya	No / Tidak	Not Applicable/ Tidak berkaitan
Newspaper/ magazines Akhbar/ majalah			
Television/ Televisyen			
Radio/ Radio			
Billboards/ Papan iklan			
Internet/ Internet			
Others? Please specify: Lain-lain? Sila nyatakan: ________________			

6. In the last 30 days, have you noticed any advertisements or signs promoting cigarettes in the following places:

Dalam tempoh 30 hari yang lalu, adakah anda melihat sebarang iklan atau papan tanda mempromosikan rokok di tempat berikut:

Places/ Tempat	Yes/ Ya	No/ Tidak	Not Applicable/ Tidak berkaitan
Stores where tobacco is sold/ Kedai di mana tembakau dijual			
Television/ Televisyen			
Radio/ Radio			
Billboards/ Papan iklan			
Posters/ Poster			
Newspapers/ magazines Akhbar/ majalah			
Cinemas/ Panggung wayang			
Internet/ Internet			
Public transportation vehicles or stations/ Kenderaan atau stesen pengangkutan awam			
Public walls/ Tembok awam			
Anywhere else?			

© 2026 Sreeramareddy C.T. et al. DOI:10.18332/tid/221104

Please specify/ Ditempat lain? Sila nyatakan ________________			

7. In the last 30days, have you noticed any advertisements or signs promoting e-cigarettes heated tobacco products like iQOS or GLO in the following:

Places/ Tempat	Yes/ Ya	No/ Tidak	Not Applicable/ Tidak berkaitan
Stores where tobacco is sold/ Kedai di mana tembakau dijual			
Television/ Televisyen			
Radio/ Radio			
Billboards/ Papan iklan			
Posters/ Poster			
Newspapers/ magazines Akhbar/ majalah			
Cinemas/ Panggung wayang			
Internet/ Internet			
Public transportation vehicles or stations/ Kenderaan atau stesen pengangkutan awam			
Public walls/ Tembok awam			
Anywhere else? Please specify/ Ditempat lain? Sila nyatakan ________________			

Indicate the extent to which you either support or oppose the current laws about tobacco control in Malaysia

	Strongly support	support	oppose	Strongly oppose	refuse	Don't know
Smoking is prohibited on public transportation, public spaces, and workplaces such as in restaurants, health, education, government, and cultural facilities; and indoor stadiums						

all forms of tobacco products advertising, promotion, and sponsorship are prohibited						
combined picture and text health warnings are required to occupy 50 percent of the front and 60 percent of the back of unit packages of cigarettes and cartons of all tobacco products						
the law prohibits the sale of tobacco products via vending machines, the internet, small packets of cigarettes, and single cigarettes						
The sale of tobacco products is prohibited for persons under the age of 18						

Indicate the extent to which you support or oppose the new international strategy (GEG) to control tobacco in Malaysia

	Strongly support	support	oppose	Strongly oppose	refuse	Don't know
**Limit nicotine**<br><br>If you could get nicotine in products other than cigarettes, would you support or oppose a law that reduces the amount of nicotine in cigarettes, to make them less addictive?						
**Ban additives**<br>Would you support or oppose a law that bans all additives, including flavoring agents such as menthol etc, in cigarettes?						
**Restrict retailers**						

© 2026 Sreeramareddy C.T. et al. DOI:10.18332/tid/221104

Would you support or oppose a law that restricts the number of places where cigarettes could be sold?						
**Ban cigarette manufacture and sales**<br><br>Would you support or oppose a law that bans the manufacture and sale of cigarettes and other tobacco products within 10 years?						
**Ban cigarette manufacture and sales with cessation support**<br>Would you support or oppose a law that bans cigarettes and other smoked tobacco within 10 years, if the government assists in helping smokers quit?						
**Ban cigarette manufacture and sales with alternate products available**<br><br>If you could get nicotine in products other than cigarettes, would you support or oppose a law that bans sales of cigarettes, but makes alternative forms of tobacco available?						

Limit nicotine

This next set of items is about possible laws that could be used to control tobacco products and tobacco companies.

Restrict retailers

The following measures have been suggested to reduce smoking. Please tell us what you think about each suggestion.

Ban cigarette manufacture and sales

© 2026 Sreeramareddy C.T. et al.
DOI:10.18332/tid/221104

The next set of items is about the possible laws that could be used to control tobacco products and tobacco companies. Would you support or oppose a law that bans the manufacture and sale of cigarettes and other tobacco products within 10 years?

Ban cigarette manufacture and sales with cessation support

The next set of items is about the possible laws that could be used to control tobacco products and tobacco companies.

Would you support or oppose a law that bans cigarettes and other smoked tobacco within 10 years, if the government assists in helping smokers quit?

Ban cigarette manufacture and sales with alternate products available

If you could get nicotine in products other than cigarettes, would you support or oppose a law that bans sales of cigarettes, but makes alternative forms of tobacco available?

The content has been provided by the author(s) and has not been reviewed, verified, or endorsed by European Publishing. It may not have undergone peer review. The views, opinions, and recommendations expressed are solely those of the author(s) and do not necessarily reflect the position of European Publishing. European Publishing accepts no responsibility or liability for any consequences arising from the use of, or reliance on, this content.

© 2026 Sreeramareddy C.T. et al.
DOI:10.18332/tid/221104
